# Supplementary material for: Are carbon emissions trading and green financial instruments synergistic? -Comprehensive quantitative research based on content analysis
Source: PLoS One. 2024 Mar 7;19(3):e0298601. doi: 10.1371/journal.pone.0298601 (PMC10919601; doi:10.1371/journal.pone.0298601)
Supplement: S1 Data — (ZIP) [file pone.0298601.s001.zip › Data/地级市Z_F工作报告.zip!/░▓╗╒/╨√│╟╩╨/└┤╫╘╣1⁄2╓┌║┼í╛┬φ┐╦╩2╛▌═°í┐_2015.docx]

一、2014年工作回顾

　　刚刚过去的一年，面对前所未有的矛盾挑战和繁重任务，全 市上下在市委的坚强领导下，认真贯彻省委、省政府的决策部署，全面落实“11331”发展思路，坚持稳中求进总基调，坚持“好为标准、快字当先”，突出重 点、攻坚克难，“六年并进”捷报频传，“六城同创”成果丰硕，发展改革稳定各项工作亮点纷呈，保持了经济稳步发展、民生持续改善、社会和谐稳定的良好局 面。

　　 ——经济运行总体稳健。主要指标增幅继续高于全省平均水 平。初步预测，地区生产总值约910亿元，同比增长9.1%左右。财政收入174.9亿元，增长10.9%；地方财政收入120.2亿元，增长 11.7%。规模以上工业增加值402.7亿元，增长11.3%。固定资产投资1140.1亿元，增长16.6%。社会消费品零售总额342.3亿元，增 长13.1%。进出口总额16.9亿美元。完成节能减排年度目标任务。荣获2013年度省政府目标管理绩效考核先进单位。宁国再次跻身“中国中小城市综合 实力百强县市”，居全省首位。

　　——质量效益不断提升。服务业增加值305亿元，占生产 总值比重提高0.3个百分点。汽车及装备制造、新材料、食品医药三大产业占规模工业比重达41.5%，提高2.9个百分点；战略性新兴产业产值占比提高 2.4个百分点；钢铁和铜加工产业占比下降4个百分点。工业利润首次突破百亿，总量、增幅均居全省第5位。工业经济效益综合指数提高5个百分点。工业投资 占固定资产投资比重达50%，制造业投资占工业投资比重提高1.4个百分点。工业化率、城镇化率分别提高0.2和1个百分点。

　　——多年期盼终于如愿。市开发区升级为国家级经济技术开 发区。成功创成省级文明城市和全省首个省级生态市，获得争创全国文明城市提名资格。顺利通过“中国文房四宝之乡”复评并更名为“中国文房四宝之城”。宣城 海关和出入境检验检疫局正式开关开检。市体育馆、图书馆、规划馆主体工程全面完成。宣城至黄山高速公路全线贯通。合福铁路宣城段主体工程全面建成。水阳江 开卡工程顺利完工。

　　 ——发展成果普惠民生。城镇居民人均可支配收入约 25000元，增长10%左右，增幅继续保持全省领先。农村居民人均可支配收入约11500元，增长11%左右。城南菜市场、向阳大道等一批项目的历史遗 留问题得到有效化解。保障性安居工程开工率、建成率均居全省前列。1013户以船为家渔民上岸安居工程全面完成，“老字号”群体工龄补助发放到位。改扩建 幼儿园开工率、竣工率均居全省第1位。公共文化场馆等级率、人均体育场地面积均居全省第3位。市区新建、改扩建中小学9所。改造提升市区菜市场5个、公厕 35座。省级卫生城市创建全面启动。建成城区道路17条，完成道路“白改黑”5条。100台新能源公交车投入使用。

　　一年来，我们主要做了以下工作：

　　（一）以提高质量效益为中心，着力推进产业转型升级。深 入推进工业突破年活动。596个项目列入省工业投资导向计划。新开工、投产、达产重点项目300个以上。新增规模企业210户，净增产值超亿元企业20 户，中鼎集团实现百亿企业零的突破。进一步理顺市开发区体制机制，宣城承接产业转移集中示范园区获省政府批复。中鼎动力铸造生产线、弘雷金属复合新材料等 一批重点项目投产。集瑞联合重工专用车、宣城海螺粉磨系统、司尔特硝基复合肥等项目开工建设。市工投公司正式运作。扎实开展文化旅游发展提升年活动。文化 产业增加值、旅游收入分别增长13.5%和18%左右。实施文化旅游产业“八大工程”三年行动计划，30个重点调度项目完成投资26.2亿元。7家企业入 选“安徽民营文化企业100强”，3家企业被命名为省文化产业示范基地，中国宣纸股份有限公司入选十大最具影响力国家文化产业示范基地。创建4A级景区2 个。“山水诗乡、多彩宣城休闲养生度假游”入选全国乡村旅游与休闲农业十大精品线路。旌德成为“中国宣砚之乡”。泾县、广德分别荣获“中国最美生态休闲旅 游目的地”和“中国最美生态休闲旅游名城”称号。敬亭山旅游度假区建设开始启动，敬亭诗径、双塔维修等项目开工建设，双广路、双塔湿地景区提升工程完工。 成功举办首届敬亭山“三月三民俗文化旅游月”系列活动。加快发展现代服务业。出台了促进健康养老服务、流通产业、电子商务加快发展等政策意见。组织编制城 市商业网点规划。市中小商贸流通企业服务平台试运行。广德成为全省唯一服务业综合改革试点县。大润发超市、星隆国际广场投入运营。双桥物流园区“三横三 纵”主干道和旅游商品博览城、联邦物流一期、亚夏汽车财富广场首批4S店建成。电子商务蓬勃发展，入选省电子商务示范园区1个，5个县电商发展指数跻身全 省县域十强。绩溪入选全省首批电子商务进农村综合示范县。

　　宁国获批全省首家阿里巴巴“千县万村”建设试点。“城市 一卡通”工程启动实施。浦发银行宣城分行试营业。徽商银行实现县域全覆盖。新设村镇银行2家。大力发展普惠金融，新建农村金融综合服务站220个，开展社 区银行建设试点。广德成立全省首家民间融资服务中心。培育壮大现代农业。粮食总产增长2.2%。落实最低收购价政策，收购粮食60万吨。宣州区荣获“全国 粮食生产先进单位”称号。农产品加工产值增长14.8%。新增省级农业产业化示范区1个、现代农业示范区2个。获批国家林业重点龙头企业3家、省级161 家。列入国家蔬菜标准园2个、省级4个。建成标准高效茶园1.2万亩。收购烟叶46万担。新增部级畜禽标准化养殖示范场1家、水产健康养殖示范场2家。新 注册农民专业合作社277家、家庭农场982家；创建国家级农民合作社示范社16家、省级9家，省级示范家庭农场24家。新增“三品”认证30个。绩溪成 功创建全国绿色食品原料（茶叶）标准化生产基地。宁国通过国家级山核桃生物产业基地评审。

　　（二）以项目建设为抓手，着力扩大有效投入。推进项目提 质提效。在建重点项目500个，完成投资620亿元，竣工项目221个；300个“调结构、转方式”重点项目完成投资420亿元。列入省“861”项目 382个，投资完成率、项目开工率和竣工率均居全省前列。谋划储备项目601个；争取中央预算内投资及省补助资金超8亿元。提升招商合作水平。完善招商工 作机制，围绕主导产业，强化精准招商，亿元以上项目到位省外资金635亿元，在全省继续保持领先；实际利用外资6.6亿美元，增长20%；引进项目产业契 合度达50%。加强与央企、知名民企合作，新签项目55个。分别与南京、杭州签署深化经济社会发展合作框架协议。成立台湾同胞投资企业协会。新塘羽绒产业 园正式落户。加大项目保障力度。市国投15亿元、市开发区建投9亿元、宁国国投13亿元企业债券成功发行。全市直接融资达45亿元，创历史新高。加大政银 企合作，新增人民币贷款108.4亿元。为335家企业办理土地抵押融资169亿元。全市组卷报批土地2.5万亩，清理“三类”土地1.26万亩。市本级 完成征地8400亩，收储国有土地8950亩，实现地票交易399亩，完成拆迁39万平方米。

　　（三）以深化改革创新为引领，着力增强内生动力。稳步推 进重点领域改革。深化行政体制改革。市级行政审批事项精简32%，实际办理时限压缩18.6%。完成非行政许可清理和前置审批条件目录清单。在全省率先公 布市级政府权力和责任清单，政府权力事项精简50.2%。建立市级涉企收费清单制度,涉企收费项目精简43%,年减轻企业负担3400余万元。完成市县工 商质监食品药品监管体制改革。深化财税体制改革。建立全口径预算体系，开展政府购买公共服务试点，邮政业和电信业“营改增”试点顺利完成。深化农村综合改 革。宣州、广德完成农村土地承包经营权确权登记颁证试点。宁国统筹城乡土地使用制度综合改革试点通过省评估验收。提升自主创新能力。新认定国家级高新技术 企业35家、国家火炬计划重点高新技术企业2家、省创新型（试点）企业10家。新批省级工程技术研究中心4家、企业技术中心5家。获省科技进步奖4项。深 化与合工大等高校产学研合作，组建皖江禽产业研究院，省民营企业科技创新联盟研发基地落户我市。主导制定省地方标准11项，居全省第2位。新增著名商标 30件、安徽名牌20个。大力发展民营经济。落实商事制度改革政策，实施新的市场主体住所（经营场所）登记管理办法，推行企业注册“一表制”和市区基本建 设项目并联审批收费。新增民营企业4610家、个体工商户16394户，完成“个转企”1029户。与省工商联成功举办“商会合作共建皖江—宣城行”活 动。民营经济增加值占生产总值比重提高1个百分点，民间投资占比超过70%。

　　（四）以建设区域中心城市为目标，着力提升城镇化水平。 加快中心城市建设。完成市级土地利用总体规划调整。新编、续编和增编城市总规等各类规划79项。完成地下管线普查。市区续建和新建城市建设项目167个， 完成投资152.2亿元，其中基础设施及公共项目投资61.4亿元。合工大宣城校区二期工程13个房建单体交付使用。宛溪河综合整治二期及周边棚户区改 造、国防动员训练基地、关庙水库等重点项目和泥河等4条河流截污工程加快推进。水阳江大道闭合段东桥工程进场施工。完成207个老旧小区整治任务。完成数 字城管二期建设。出台《打造皖苏浙交汇区域中心城市促进房地产业健康发展意见》，促进人口向中心城区集聚，商品房销售突破百万平方米。加快交通建设会战。 完成交通建设投资86亿元，高速公路投资占全省近四分之一。新增高速公路76公里、一级公路55公里。完成国省道路面改善128公里，公路路况指标进入全 省前四名。宣城、郎溪客运总站开工建设，宁国长途汽车站基本建成。芜申运河郎溪段及定埠港主体工程基本完工。改善县乡公路路面125公里，改造农村公路危 桥62座。杭黄铁路绩溪段开工建设，皖赣铁路芜宣段扩能改造有序推进。完成水利建设投资6.9亿元。郎溪中斗闸、双桥联圩城市规划区堤防除险加固工程、城 东联圩宛溪河治理一期工程完工。完成电网建设投资比上年翻一番。光纤入户项目快速推进，4G网络实现全覆盖。深入开展“六城同创”。文明市创建全面启动。 市区集中整治“六乱”行动取得实效。宁国跻身全国县级文明城市提名城市。完成国家环保模范城市创建省级预评估。郎溪、泾县通过国家生态县技术评估。新增国 家生态乡镇17个。建立大气污染防治工作体系，大气环境质量优良率达92.3%，比上年提高4.4个百分点。实行最严格水资源管理制度，开展江河沿线环境 专项治理、非法采砂联合执法和环保专项行动，城市集中饮用水水源地和国控断面水质达标率均为100%。扎实推进国家园林城市和森林城市创建，新建城市公园 5个，新增绿化面积175万平方米；新造林18万亩，广德、绩溪成功创建省级森林城市，创建森林城镇和村庄92个。加快美好乡村建设。编制完成县市区村庄 布点规划。35个省级重点示范村通过验收，57个省级中心村建设扎实推进。郎溪成为全省美好乡村建设先进县。旌德实现中心村建设全覆盖。新增中国最美休闲 乡村2个、全国休闲农业和乡村旅游三星级以上示范点5个。“三线四边”、矿山生态环境得到有效整治，10条示范带建设水平进一步提升。农村清洁工程扎实推 进，75个乡镇、522个村实行垃圾处理市场化，63个乡镇、352个村开展垃圾分类减量处理工作。

　　（五）以民生优先为原则，着力保障和改善民生。大力实施 民生工程。省定33项民生工程和新增公交线路等13项市政府民生实事年度任务全面完成。城镇新增就业4.9万人，城镇登记失业率低于省控指标1个百分点。 城乡基本养老保险、城镇基本医疗保险基本实现全覆盖。实现廉租房、公租房并轨运行，将进城落户农民纳入住房保障体系，全市住房保障总户数达5.4万户。完 成9327户农村危房改造工程。城市、农村居民低保标准分别提高7%和10.4%。农村“五保”集中供养率稳步提升。全面发展社会事业。完成244所义务 教育学校标准化建设任务。宣州、广德、宁国通过国家义务教育基本均衡县评估认定。高考本科达线率首次达60%。参加省运会获金牌数增加近30枚，位次前移 四位。参加省少数民族运动会取得历史最好成绩。成功举办市第二届残疾人运动会。积极推进“中国书法城”、“中华诗词之乡”争创工作，“书法教育进课堂”活 动有效开展。绩溪手龙舞和郎溪跳五猖被列入国家级非遗名录。村级文化广场建设通过文化部中期督查。郎溪、广德、宁国被列为首批省级公共文化建设示范区。5 个省级农民文化乐园示范村建设任务完成。基层医改、县级公立医院综合改革巩固完善，基本公共卫生服务水平得到提升。市人民医院改扩建一期工程、残疾人康复 中心开工建设。稳妥实施“单独两孩”政策，出生人口政策符合率、性别比居全省前列。深入开展扶贫攻坚，建立单位包村、干部包户制度，扶贫济困工作得到加 强。宁国云梯畲族乡千秋文化园成为全国民族团结进步教育基地。第三次全国经济普查工作获得国家表彰。工会、共青团、妇女、儿童、老龄和红十字、慈善福利事 业迈出新步伐。外事侨务、民族宗教、档案、地方志、人民防空、防震减灾、烟草专卖等工作继续加强。加强和创新社会治理。启动社会服务管理信息化建设。宁国 在全省率先建成县级社会管理服务中心。圆满完成第九届村委会换届选举工作。深入推进平安宣城、法治宣城建设，荣获全国“六五”普法中期先进市称号。司法行 政公共法律服务工作覆盖到村。在全省率先开设交通事故救治“绿色通道”。实施市领导担任市信访局“第一局长”工作制度，领导干部开门接访工作延伸到乡镇， 信访形势总体平稳。调整应急管理体制，加强应急预案编制和演练。深入开展安全生产“六打六治”专项行动，事故起数、死亡人数持续下降。推行首席质量官制 度。启动省级食品安全示范县创建，实现食品生产环节监管全覆盖。启动“全国双拥模范城”争创工作，优抚安置工作水平不断提升。

　　 (六) 以教育实践活动为契机，着力提升政府服务效能。继续优化政风。深入开展党的群众路线教育实践活动，查摆突出问题142项，解决“老字号”等具体问题16 项，建立完善相关制度35项。严格执行中央八项规定、国务院“约法三章”和省市委“三十条规定”，认真践行“三严三实”要求，聚力整治“四风”和“小四 风”问题。整改清退农民负担违规资金1525.4万元。市本级清理腾退超标办公用房2.5万余平方米，整改工作得到中央领导批示肯定。加强法治政府建设。 按照党的十八届四中全会精神要求，认真开展依法治市工作，自觉接受人大监督，依法执行人大决议决定，主动接受政协民主监督。市政府负责同志领衔办理市人代 会议案、人大代表建议和政协委员提案共19件。评定第一批依法行政示范单位。出台重大行政决策程序规定，制定“四项制度”，完善规范土委会、规委会等制 度。强化政务公开，出台加强政府信息公开工作意见，坚持开展“百姓问政”、在线访谈等政民互动活动，推进政务微博。强化勤政廉政。严格落实政府系统党风廉 政建设责任制。公开市直部门预决算和“三公”经费信息，“三公”经费支出下降31.1%。建立领导干部工作日志制度，强化政务督查和效能监察，深化民主考 评和政风行风评议。理顺市区公共资源交易管理体制和运行机制，建立市区统一的管理机构和平台。扩大审计覆盖面，加大对领导干部经济责任、政府建设资金审计 力度，节约资金3.7亿元。

　　各位代表，过去的一年，在新挑战新困难持续增加的情况 下，取得这些成绩实属不易。这是省委、省政府和市委坚强领导的结果，是市人大、市政协监督支持的结果，更是全市人民团结拼搏的结果。在此，我代表市人民政 府，向全体人大代表、政协委员，向全市广大工人、农民、知识分子、干部、离退休老同志，向驻宣部队、武警和消防官兵、政法干警，向各民主党派、工商联、无 党派人士、各人民团体，向所有关心、支持、参与宣城建设发展的同志们、朋友们，表示崇高的敬意和衷心的感谢！

　　过去一年迈进的征程中，既仍有亟待解决的老问题，又遇到 新的困难和挑战：在经济下行压力加大、约束加剧的环境下，经济增长速度整体回落，特别是部分指标未能实现市三届人大五次会议确定的目标；传统产业产能过 剩，新兴产业比重较小，服务业发展滞后，结构不优的矛盾进一步凸显；资源要素供求关系紧张，企业生产经营困难加剧；财政收入增长放缓，房地产市场低迷，政 府债务压力增大；市本级不大不强、县域发展不平衡现象仍较突出；环境保护、安全生产、食品药品安全等方面还存在一些突出问题；少数公务员缺乏担当、能力不 足，“四风”问题仍然存在。对此，我们必须高度重视，切实加以解决。

　　二、2015年工作目标和重点任务

　　2015年是全面深化改革的关键之年、全面推进依法治市 的开局之年，也是全面完成“十二五”规划的收官之年。做好今年政府工作，责任和压力更大，机遇与挑战并存。必须科学看待新常态。正确认识经济发展新常态下 速度变化、结构调整、动力转换的基本特征，准确把握新常态下实现新发展的根本取向、基本路径和原则遵循，自觉按照“八个更加注重”要求谋划发展。必须准确 把握新常态。紧紧抓住国家实施长江经济带战略等重大历史机遇，坚持发展第一要务，扩大多方面对外合作，充分利用新一轮科技革命和产业变革提供的宝贵机会， 不断拓展健康养老、文化旅游等消费热点持续升温所蕴含的巨大增长空间，进一步发挥我市良好的生态优势，顺势而为、乘势而上，促进经济社会转型发展。必须主 动适应新常态。坚持有质量、有效益、可持续的发展，全面深化改革开放，更加突出创新驱动，以有效的科学举措推动新常态下的新发展，确保“十二五”圆满收 官，为在“十三五”全面建成小康社会奠定坚实的基础。

　　今年政府工作的总体要求是：全面贯彻党的十八大和十八届 三中、四中全会及中央、省、市经济工作会议精神，以提高经济发展质量和效益为中心，以建设皖苏浙交汇区域中心城市为主线，坚持“好为标准、快字当先”，稳 中求进，落实“11331”发展思路不变，推进“六年并进”、“六城同创”不变，统筹做好稳增长、促改革、调结构、惠民生、防风险的各项工作，努力实现产 业提升、城市提升、交通提升、环境提升、民生提升，促进经济社会持续稳定健康发展。

　　建议全市经济社会发展的主要预期目标是：地区生产总值增 长8.5%左右；规模以上工业增加值增长11%左右；服务业增加值增长8%左右；财政收入增长8%左右；固定资产投资增长13%左右；社会消费品零售总额 增长13%左右；进出口总额增长3%左右；城镇和农村居民人均可支配收入增幅高于经济发展水平；确保完成“十二五”节能减排任务。

　　 为实现上述目标，要着力做好以下六个方面重点工作：

　　（一）促进产业转型升级，增强中心城市经济实力

　　大力实施“工业强市”战略，加快建设新兴制造业基地。聚 焦市本级工业发展。用好国家级开发区品牌，支持市开发区开展“项目攻坚大会战”，加快培育发展主导产业，确保签约项目80个，新开工、竣工工业项目22个 和20个，确保主要经济指标增幅高于全市平均水平。市财政再注入5000万元，做大市工投公司和担保公司规模。加快推进中鼎动力新型发动机、集瑞联合重工 专用车、司尔特硝基复合肥、宣城海螺粉磨系统等重点项目建设，确保国投宣城电厂二期等项目建成投产。继续加强与央企、知名民企对接，力争特种车辆工程技术 研究院等项目落户我市。开工建设省民营企业科技创新联盟研发基地。深入实施“千百万”工程。重点推进汽车及装备制造、新材料、食品医药产业发展，力争实现 产值占比达45%左右。实施“双百”重点工程，新开工、投产项目各100个，确保工业投资增长13%以上。加强重点企业培育，力争实现产值超10亿元25 户、超亿元360户，新增规模以上企业160户。发展壮大民营经济，新注册民营企业3000户、个体工商户10000户以上。加强企业家队伍建设，大力弘 扬企业家精神。加快推进创新驱动。力争新认定高新技术企业25家、省创新型试点企业4家，新建省级工程技术研究中心4家、争创国家级1家，力争战略性新兴 产业、高新技术产业产值分别增长13%和14%左右。深化产学研协同创新，加强知识产权保护与应用，新增发明专利授权120件。推进省级“两化融合”示范 企业建设。争创省级工业设计中心2家。深入实施商标发展和质量强市战略，加大企业商标和名牌产品培育力度。

　　突出发展文化旅游、健康养生产业，加快建设国际性旅游目 的地城市。确保文化产业增加值、旅游收入分别增长10%和15%以上。打造文化旅游龙头。强力推进敬亭山旅游度假区5A景区创建，加快环山南路、北路和敬 亭诗径、石涛文化园、韩愈文化园、中华诗词文化博览园、太白文化民俗村改造、林相改造提升等项目建设。举办第七届中国文房四宝文化旅游节和第二届敬亭山 “三月三民俗文化旅游月”系列活动。积极推进安徽国际旅游商品直销中心建设。加大文教创意产业园区项目建设和招商力度。促进全域文化旅游发展。落实皖南国 际文化旅游示范区建设规划纲要，制定行动计划和促进文化旅游产业改革发展的政策措施，加快实施文化旅游产业“八大工程”，推进28个重点项目建设。加强旅 游景区和“智慧旅游”建设，创建4A级以上景区2—3家，支持太极洞争创5A级景区，力争龙川国家风景名胜区获批。培育和扶持文化旅游龙头企业发展。加强 宣传营销，强化与长三角地区文旅联动。推进带薪休假基地和中小学生研学旅行基地建设。以建设面向长三角的健康养生基地为重点，以打造全国健康养生示范城市 为平台，加快发展健康休闲养生养老产业。

　　提升发展商贸流通服务业，加快建设区域性商贸物流中心。 实施城市商业网点规划。科学规划、整体开发，着力打造中央商务区、城市特色街区等。充分发挥中小商贸流通企业服务中心的服务和引导作用，加快限上商贸企业 培育。积极争取国家城市物流配送试点，大力推进流通产业发展。加快双桥物流园区基础设施建设，加大市场培育力度，力争新塘羽绒产业园建成投产，确保联邦物 流、冷链物流、林泰物流等项目投入运营。搭建电商服务平台，建设电商服务中心，壮大电商主体，促进电商产业集聚发展。推进电子商务进农村试点，探索跨境电 商试点。

　　积极发展现代农业，加快建设面向长三角的优质农产品供应 基地。保持粮食稳定增长。继续做好粮食最低价收购工作。加快林特产品、中药材等特色农产品基地建设。积极构建现代烟草农业模式。稳步提升茶产业。大力推进 油茶产业致富工程和竹产业提升工程。新建1000亩市区保障性蔬菜基地，争创4个省级以上蔬菜标准园。推进畜禽标准化示范养殖小区和水产健康养殖示范场创 建。加快现代农业示范区建设，积极发展农产品精深加工，力争加工产值增长12%以上。培育壮大新型农业经营主体，加快土地流转步伐，积极创建国家级、省级 示范社（场）。大力发展农业社会化服务，提高农技推广和农业机械化水平。扩大农业保险覆盖面。加快实施皖南特色农业气象服务工程。强化农产品质量安全和农 业投入品监管，确保市农产品质量安全检验检测中心投入运行。加大农产品品牌建设力度。新增“三品一标”认证数30个以上。大力实施“放心粮油”和“主食厨 房”工程，切实做好危仓老库维修改造工作。

　　（二）加快城乡基础设施建设，增强中心城市综合承载力

　　完善中心城市功能，塑造宜居宜业宜游城市特色。完成新一 轮城市总体规划、环南漪湖地区概念性总体规划、敬亭山风景名胜区重点地段详细规划等修编，开展港口布局、宁宣产业园等专项规划研究。出台城市控制性详细规 划通则。加快市区164个项目建设，确保完成城市建设投资120亿元。续建、新改建77条城市道路，开工建设宛陵路跨宛溪河大桥、青弋江大道跨皖赣铁路立 交桥、宛陵湖西湖河叉工程和宛陵湖东湖等项目，加快北门棚户区改造、宛溪河综合整治二期及周边棚户区改造、合工大宣城校区三期、国家宣纸及文房用品质量监 督检验中心等项目建设，确保市图书馆、规划馆、体育馆、示范性综合实践基地和国防动员训练基地等投入使用。实施并完成谢朓楼维修工程。稳妥推进彩金湖新区 建设。启动档案馆和阳德路改造、文景路、水阳江大道闭合段北桥等项目。力争开工建设市体校、游泳馆。做好科技场馆项目前期工作。

　　优化城镇布局，促进城镇协调有序发展。完成县城总体规划 修编和全国重点镇总体规划编制工作，优化完善城乡空间布局，加快构建布局合理、联系畅通、优势互补、协调发展的市域城镇体系。增强县城的区域带动和辐射作 用，推进12个全国重点镇建设，加快实现“工业向开发区集中、人口向重点城镇集中、农村基本公共服务向中心村以上社区集中”，实现资源的集约化利用。

　　加强美好乡村建设，打造“美丽中国”建设先行区。坚持 “点、线、面”同步推进。巩固92个省级中心村建设成果，抓好今年55个省级中心村建设，打造10条左右美好乡村精品旅游线路。巩固农村清洁工程市场化改 革成果，推进农村卫生保洁常态化和垃圾分类减量处理。加强涉农项目和资金整合，完善村民自治和群众参与机制，健全美好乡村建设长效管护制度。

　　深化交通建设会战，加快形成区域性交通枢纽。继续开展交 通建设会战年活动，建设项目80个以上，在建公路里程超过800公里，完成投资突破80亿元。推进宣狸、溧广等6条高速公路建设，开工建设宁绩高速鸿门互 通工程，宁千、宣南铜高速完工，新增高速公路里程77公里。推进11条一级公路建设，完成S322宣泾和G205泾南一级公路建设，新增一级公路80公 里。完成国省道和县乡公路路面改善各100公里。完成芜申运河郎溪段及定埠港建设，加快水阳江航道整治工程和宣州综合码头项目建设。确保郎溪客运总站建成 使用，完成宣城客运总站主体工程，开工建设绩溪客运站。改造农村公路危桥25座。全力保障合福铁路开通运营。做好皖赣铁路扩能改造工程芜湖至宣城段、杭黄 铁路征迁服务工作，推进宣城火车站建设。开工建设商合杭铁路，力争皖赣铁路扩能改造工程宣城至绩溪段开工建设。推进巷口桥铁路物流中心等项目。

　　加强水利、电力、通讯等基础设施建设，夯实城乡发展的保 障条件。全面完成关庙水库、清湘湖水库和宛溪河城区段防洪、敬亭圩除险加固等重点工程建设，力争开工水阳江生态蓄水工程、城东联圩济川段除险加固、青弋江 治理等项目。实施中小河流治理项目，启动水阳江中游综合治理项目前期工作。持续抓好农村饮水安全工程和“八小工程”建设。续建、新建12个110千伏以上 主电网工程，竣工投产10个。加快宣州区配电网网架建设和县域农网升级改造。启动准东至华东（皖南）特高压换流站工程前期工作。加大全光网络集镇和行政村 建设。完成新一代多普勒天气雷达塔楼建设。

　　（三）全力扩大有效投入，增强中心城市发展动力

　　着力项目提质提效。高质量开展“十三五”规划编制工作， 谋划储备重点项目500个以上，力争纳入国家、省“十三五”规划的项目数不少于“十二五”；争取国家、省补助资金不少于上年。实施重点项目400个以上， 确保列入省亿元以上重点项目年度投资完成比率保持全省前列。落实最严格的耕地保护和节约集约用地制度，确保“十二五”期间单位生产总值建设用地下降 29%。认真开展永久性基本农田划定和闲置低效用地清理处置工作。加大土地报批和征迁力度。

　　切实推进招商合作。积极采用PPP等模式，推出一批社会 资本参与建设营运的示范项目。科学统筹招商引资工作，完善招商引资工作机制。围绕主导产业发展，明确主攻方向，细化招商目标企业导引图，增强招商工作针对 性和实效性。突出专业招商力量建设，积极探索市场化招商，扩大委托招商。加大与中介机构、重点商会、行业协会合作，强化以商招商。规范招商引资行为，优化 完善投资发展环境，扩大与境内外多种形式的合作，确保新引进重大项目55个，亿元以上项目实际到位省外资金增长15%左右，实际利用外资增长10%。

　　加快推进社会融资。确保社会融资规模增幅高于全省水平， 力争直接融资超过上年规模。积极争取发行地方政府债券。大力发展普惠金融。实现村镇银行县域全覆盖。加强银政担合作，提升小额贷款公司、融资性担保机构发 展水平。积极争取国开行棚户区改造资金，争取省级资金对国有融资性担保公司注资。力争首发上市企业2家，新三板挂牌企业超过3家。强化金融产品和小微企业 还款方式创新，加强银行间市场融资、应收账款融资、商标质押融资等工作。继续推进“城市一卡通”工作。强化社会信用体系建设，优化金融生态环境。加强对财 金工作统筹，防范和化解金融风险。

　　（四）深化改革开放，增强中心城市发展活力

　　以深化改革激发发展活力。深化行政体制改革。进一步理顺 市与宣州区、市开发区管理体制机制，促进一体化发展。全面完成新一轮市县政府机构改革和职能转变。继续深化行政审批制度改革，精简和调整行政审批事项，提 高审批效率和服务质量。深化预算管理制度改革，完善市与宣州区、市开发区财税体制。做好生活服务等行业“营改增”扩围工作。激发市场主体活力。深化商事制 度改革，落实企业登记改革举措，全面推行“三证合一”。继续加大非行政许可、前置审批条件清理，实行目录清单管理。市县两级全面实施涉企收费清单制度。研 究制定企业投资准入负面清单。制定公共资源交易监督管理意见，编制公共资源交易目录，完善招投标监管工作。深化农村综合改革。力争全面完成农村土地承包经 营权确权登记颁证工作。扩大农房登记发证试点范围，稳妥推进农房抵押流转。开展农村集体经济组织产权制度改革试点。加快推进供销合作社综合改革。深化宁 国、宣州、广德农村土地使用制度改革试点，积极申报国家级改革试点。推进户籍制度改革。完善户口迁移政策，建立统一的城乡户口登记制度。

　　以开放合作拓展发展空间。加强与长三角区域合作发展，主 动对接合肥都市圈、杭州都市圈，重点落实与南京合作框架协议，推进宁宣产业园前期工作，强化与区域内各城市的交通和公共基础设施、旅游、教育、医疗等领域 合作。继续实施“双百”工程，培育壮大外贸企业。发挥宣城海关、出入境检验检疫局等服务平台作用，引进外贸综合服务企业，为企业提供通关、报检等便利化条 件。推进食品农产品出口示范基地建设，启动市本级保税物流仓库项目。鼓励企业“走出去”，参与国际合作和竞争，拓展国际市场。

　　（五）加强生态文明建设，增强中心城市魅力

　　开展国家生态市创建攻坚。申报国家生态市和环保模范城市 技术评估。加强城镇园林绿化建设，力争通过国家园林城市创建验收。深入实施森林增长工程，新造林9.1万亩，建设森林示范长廊30公里；力争创成国家森林 城市，郎溪、泾县、旌德成为省级森林城市。深入开展爱国卫生运动和环境卫生综合治理，加强病媒生物防治和城市卫生基础设施建设，争取通过省级卫生城市考核 评估。

　　加强环境保护和节能减排。加大规划环评与项目环评力度， 严格项目审批准入，强化建设项目“三同时”制度落实。推行市政府委派环保督查员制度。继续开展环保“绿剑”行动，严厉打击环境违法行为。深化水生态文明城 市建设试点。坚持最严格的水资源管理，强化河道采砂监管。加大水污染防治工作力度。持续开展大气污染综合防治，加快淘汰2005年以前注册的黄标车和老旧 车辆，强化秸秆禁烧工作和高污染燃料禁燃区环境管理。推广节能环保新设备、新工艺、新技术，鼓励生产和使用节能节水节材产品。继续推进污水处理厂建设，加 快污水管网、截污设施建设，确保按期建成、正常运行。

　　不断提升城市文明程度。以文明市创建统揽城乡文明建设， 积极争创全国文明城市，支持宁国、绩溪创建全国文明城市（县城）。持续推进“三线四边”环境治理提升行动和“三车”整治工作。深化城市网格化管理。实施市 容提升工程，探索建立老旧小区综合管理新模式，整治沿街建筑立面，加强市区公厕、停车场建设与管理。开展垃圾分类试点，促进垃圾资源化利用。加大城区烟花 爆竹禁放工作力度。推进社会主义核心价值观“四大工程”，提升群众文明素质。积极推动军民融合发展，深入开展国防教育工作，全面落实优抚安置政策，合力争 创“全国双拥模范城”。

　　（六）保障改善民生，增强中心城市凝聚力

　　 加强就业服务和社会保障。深化省级创业型城市创建，培 养、引进技能型人才，加强就业创业工作，新增城镇就业岗位3.3万个。认真落实国家机关事业单位社会保险改革政策和社会保险关系转移接续政策。推进城乡居 民养老保险工作，完善被征地农民社会保障政策。规范职工医疗、工伤、失业保险市级统筹工作。全面实施城镇居民大病保险。稳步提高农村五保、优抚对象、城乡 低保等保障水平。完善“救急难”工作发现机制，加强困境儿童、城乡“三无”特困人员供养和流浪乞讨人员临时救助。启动市救助管理站建设。加快建立新型城乡 养老服务体系，推进农村敬老院转型升级，建立政府为居家养老购买服务补贴制度。促进残疾人等弱势群体家庭增收。实施精准扶贫，加大扶贫济困工作力度。促进 房地产业稳定健康发展。探索多渠道住房保障方式，提高棚改货币化安置比例，全面完成住房保障目标任务。扩大住房公积金制度覆盖面，完善提取和使用政策。认 真办好民生新实事。

　　优先发展现代教育。深入实施教育三个“三年行动计划”。 新建、改扩建幼儿园各6所，推进幼儿园公建民营试点。市区新建、续建小学8所，新建、改扩建中学5所，新增小学、初中起始年级学位1080名和300名。 全面完成义务教育学校标准化建设规划。加强师资队伍建设。扎实推进宣城中学打造“皖南名校”工程。加快宣城职教联盟发展，加强中职学校建设，推动集团化办 学和校企合作。支持合工大宣城校区、宣城职业技术学院加快发展。鼓励民间资本投入教育事业，规范发展民办教育。

　　全面发展社会事业。强化基层医改和县级公立医院改革措施 落实。完善主城区两级卫生网络。继续开展乡镇卫生院规范化管理和二级医院帮扶提升中心卫生院能力建设活动，加强农村医疗卫生三级服务网络和社区卫生服务中 心建设。加快市人民医院改扩建工程和市精神病院新院建设。完成第一次全国可移动文物普查工作，加强皖南花鼓戏等文化遗产保护和传承。加快村级文化广场、公 共电子阅览室、广电“村村通”工程建设。完成省级第三批农民文化乐园试点村建设任务。推进村级农民体育健身工程和县级国民体质监测站建设。积极开展丰富群 众生活的文娱活动，举办全市首届运动会。加强新媒体建设，推动宣城日报、宣城广电全媒体融合发展。稳妥实施“单独两孩”生育政策，加强人口计生基层基础工 作，落实奖励优待政策，确保人口计生工作继续保持全省领先。继续开展民族团结进步创建活动，依法管理宗教事务。加强统计基层基础工作，提高政府统计公信 力。支持和推进人民团体广泛参与社会管理和公共服务，扎实做好外事侨务、对台事务、防震减灾等工作，全力落实妇女和儿童“两个发展规划”中期目标任务，加 快发展老龄、残疾人和红十字、慈善福利事业。

　　加强和创新社会治理。加快建设市政务云计算中心、数据交 换平台，扎实开展社会服务管理信息化建设，推进智慧城市建设，提高社会治理水平。加强法治宣传教育，积极争创全国“六五”普法先进城市。推进公共法律服务 体系建设，扩大法律援助范围，加强社区矫正、仲裁工作。深化基层综治维稳信访工作中心（站）建设，完善矛盾纠纷排查调处工作机制，全面推进行业性、专业性 调解组织建设。加大信访工作制度改革力度，实行访诉分离，推进网上信访。深化领导干部接访下访，完善联合接访中心运行机制，建立健全司法调解、人民调解、 行政调解有效衔接机制，依法规范信访秩序，全力化解信访积案。全面完成第三届城市社区居委会换届选举工作。

　　强化公共安全防控。全面落实安全生产“党政同责一岗双责 齐抓共管”责任体系，强化安全生产监管和隐患排查治理，减少和防范安全生产事故发生。实施食品药品放心工程和检测能力提升工程，加强省级食品安全示范县创 建，继续开展“四品一械”市场安全整治。强化治超工作。深化平安宣城建设，完善社会治安防控体系，完成社会治安视频防控系统二期工程建设，依法打击违法犯 罪活动，严厉打击逃废债务、欺行霸市等不法行为。推进人民防空与经济社会融合发展。整合应急指挥平台体系，加强应急队伍建设和演练，完善应急管理体制和应 急处置联动机制。强化网络安全管理。

　　三、加强政府自身建设

　　面对新常态新挑战，必须按照“为民、务实、清廉”的总要求，依法履行政府职责，创新管理方式，提高服务效能，努力建设法治政府和服务型政府。

　　坚持宪法为上，严格依法行政。依据宪法和法律行使权力。 坚持法定职责必须为、法无授权不可为，全面推行政府权力清单、责任清单制度。健全重大决策合法性审查和风险评估机制，完善行政决策法定程序和政府法律顾问 制度，建立健全决策咨询制度，扩大公众参与度。坚持重大事项向市委报告制度，自觉接受人大的法律监督、工作监督和政协的民主监督。坚持市长领衔办理制度， 提高人代会议案、人大代表建议和政协委员提案办理质量。主动听取各民主党派、工商联、无党派人士和人民团体的意见，广泛接受社会公众和新闻舆论监督。推进 依法行政示范单位创建，强化行政执法责任制落实，提高行政执法水平。加强行政复议工作。深入推进政务公开，加大重点领域政府信息公开力度，让人民监督政 府、让权力在阳光下运行。

　　 坚持发展为先，提升服务效能。始终把加快发展作为“第一 要务”，用发展凝聚人心、破解难题、促进和谐。牢记为民宗旨，以百姓心为心，解决群众困难，谋求群众福祉。完善政务服务体系，深入推进政务服务标准化建 设。加强乡镇（街道）为民服务中心及村级代办点建设。强化企业帮办服务和跟踪服务。深化民主考评和政风行风评议活动。继续推进政民互动工程，加强“百姓问 政”、政务微博、网络政民互动等平台建设，整合优化群众网络诉求回应和“市长热线”办理平台。

　　坚持责任为重，弘扬担当精神。严格执行分管负责、牵头负 责、限时办结和责任追究“四项制度”，强化行政监察和政务督查，向为官不为“亮剑”，用铁拳整治懒政怠政行为。坚持以目标倒逼进度、以督查强化落实，推动 政府工作提速提效。大力弘扬“钉钉子”精神和当代宣城干部精神，以踏石留印、抓铁有痕的狠劲，强化担当、勇于创新，面对困难不退缩、遇到矛盾不回避，不断 创造经得起实践、群众和历史检验的业绩。

　　坚持实干为要，切实转变作风。巩固群众路线教育实践活动 成果，严格落实中央八项规定和省市委“三十条规定”，践行“三严三实”，持续整治“四风”，推进作风建设常态化、长效化。坚持求真务实，坚持问题导向，深 入基层听民声、察民情、解民忧。进一步清理检查评比活动，精简会议文件，改进调查研究和会风文风，减少往来应酬，把精力集中在加快宣城发展、保障改善民生 上。

　　 坚持清廉为本，做到“一岗双责”。全面落实从严治党要 求，严格落实党风廉政建设主体责任制度，切实加强廉政建设。扩大预算执行、经济责任、政府投资建设项目的审计覆盖面，强化民生资金使用、工程招投标、土地 出让、矿产资源开发等重点领域的监督管理。推进政府预决算公开。实施公务用车改革，规范办公用房日常管理，严格控制“三公”经费，自觉过“紧日子”，让群 众过“好日子”。加大廉政建设巡查力度，严格执行权力公开运行、廉政风险防控等制度，完善纠错问责机制，坚决纠正损害群众利益的不正之风，严厉查处各类违 纪违法行为。
